# Supplementary material for: Electrospun Polyvinylpyrrolidone-Based Dressings Containing GO/ZnO Nanocomposites: A Novel Frontier in Antibacterial Wound Care
Source: Pharmaceutics. 2024 Feb 22;16(3):305. doi: 10.3390/pharmaceutics16030305 (PMC10976148; doi:10.3390/pharmaceutics16030305)
Supplement: Supplementary file 1 [file pharmaceutics-16-00305-s001.zip › pharmaceutics-2857531-supplementary.pdf]

Supporting Information

Electrospun Polyvinylpyrrolidone-Based Dressings Containing GO/ZnO Nanocomposites: A Novel Frontier in Antibacterial Wound Care

Cristina Martín [1,*], Adalyz Ferreiro Fernández [2], Julia C. Salazar Romero [1], Juan P. Fernández-Blázquez [3], Jabier Mendizabal [4], Koldo Artola [4], José L. Jorcano [1,5] and M. Eugenia Rabanal [2,*]

[1] Department of Bioengineering, Universidad Carlos III de Madrid, 28911 Leganés, Spain; 100356873@alumnos.uc3m.es (J.C.S.R.); jjorcano@ing.uc3m.es (J.L.J.)
[2] Department of Materials Science and Engineering and Chemical Engineering & IAAB, Universidad Carlos III de Madrid, 28911 Leganés, Spain; adferrei@ing.uc3m.es (A.F.F.)
[3] IMDEA Materials Institute, 28906 Getafe, Spain; juanpedro.fernandez@imdea.org (J.P.F)
[4] Domotek Ingeniería Prototipado y Formación S.L., 20003 San Sebastián, Spain; jabier@domotek.es (J.M.); info@domotek.es (K.A.)
[5] Instituto de Investigación Sanitaria Gregorio Marañón, 28007 Madrid, Spain (J.L.J.)
* Correspondence: cristima@ing.uc3m.es (C.M.); eugenia@ing.uc3m.es (M.E.R.)

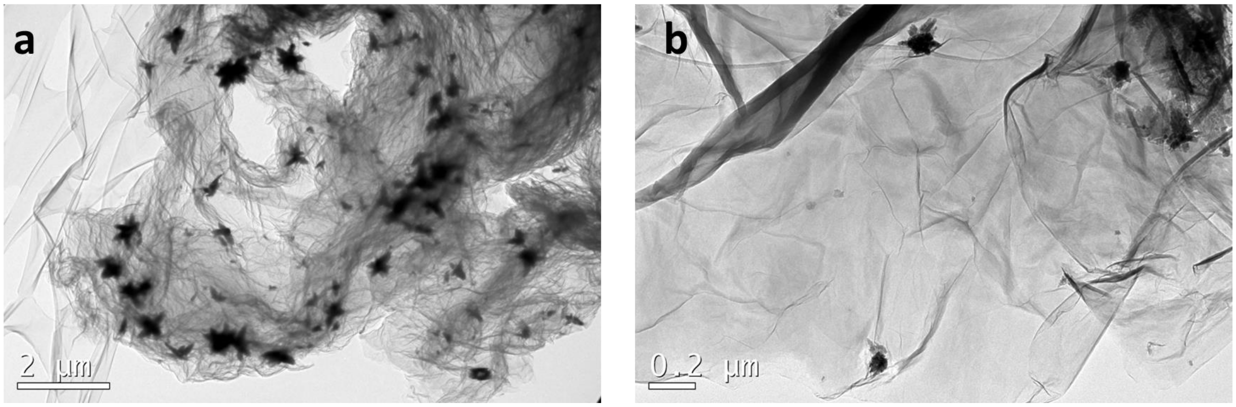

Figure S1. Representative TEM images of (a) GO/ZnO_1:1 and (b) GO/ZnO_2:1 nanocomposites.

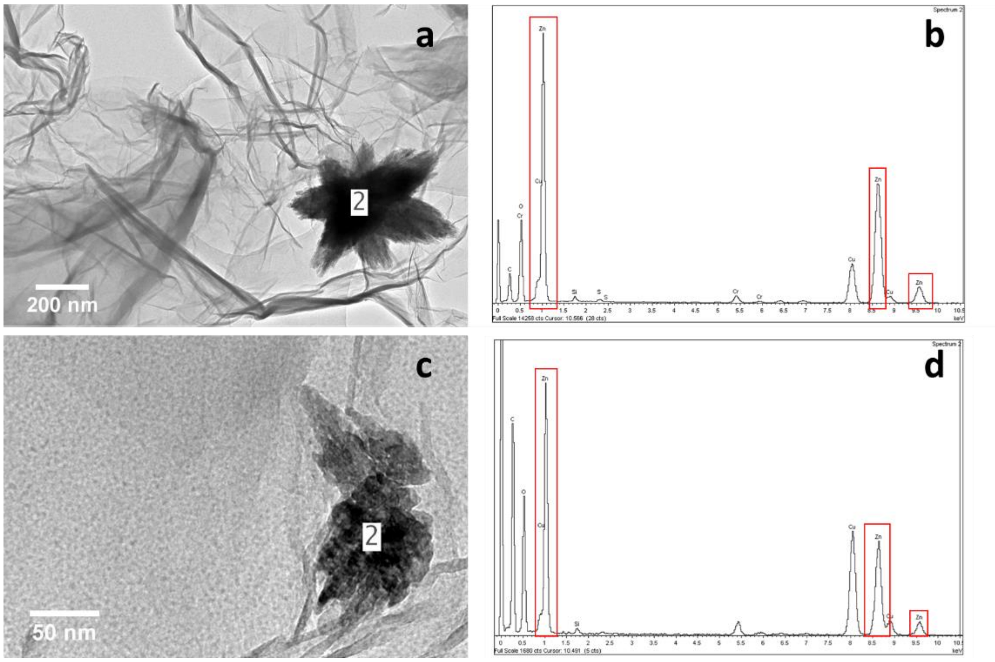

Figure S2. EDX analyses of ZnO nanoparticles on the (a,b) GO/ZnO_1:1 and (c,d) GO/ZnO_2:1 nanocomposites. Red squares indicate the peaks corresponding to Zn.

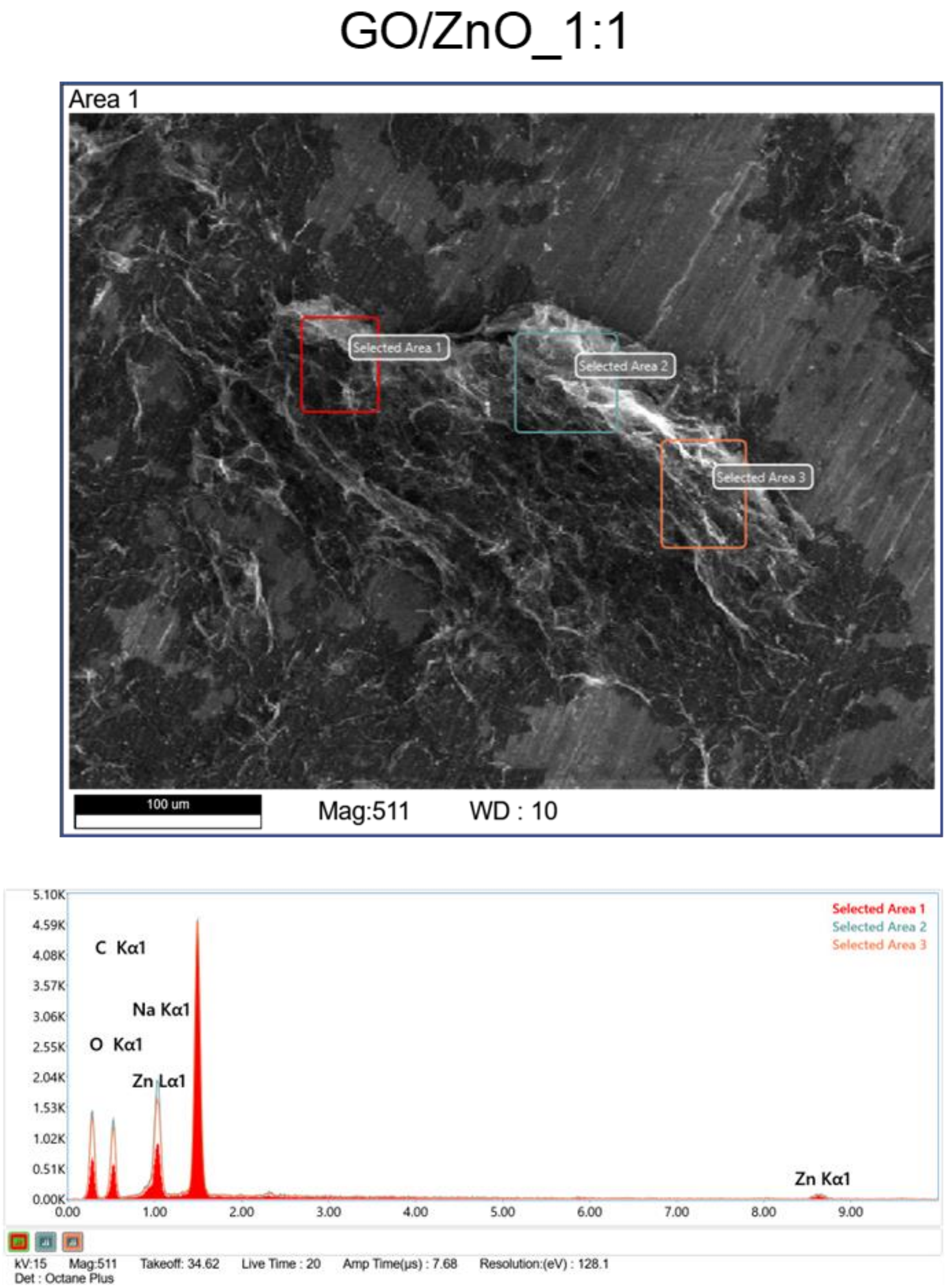

Figure S3. SEM pictures and element mapping results of GO/ZnO_1:1 composite.

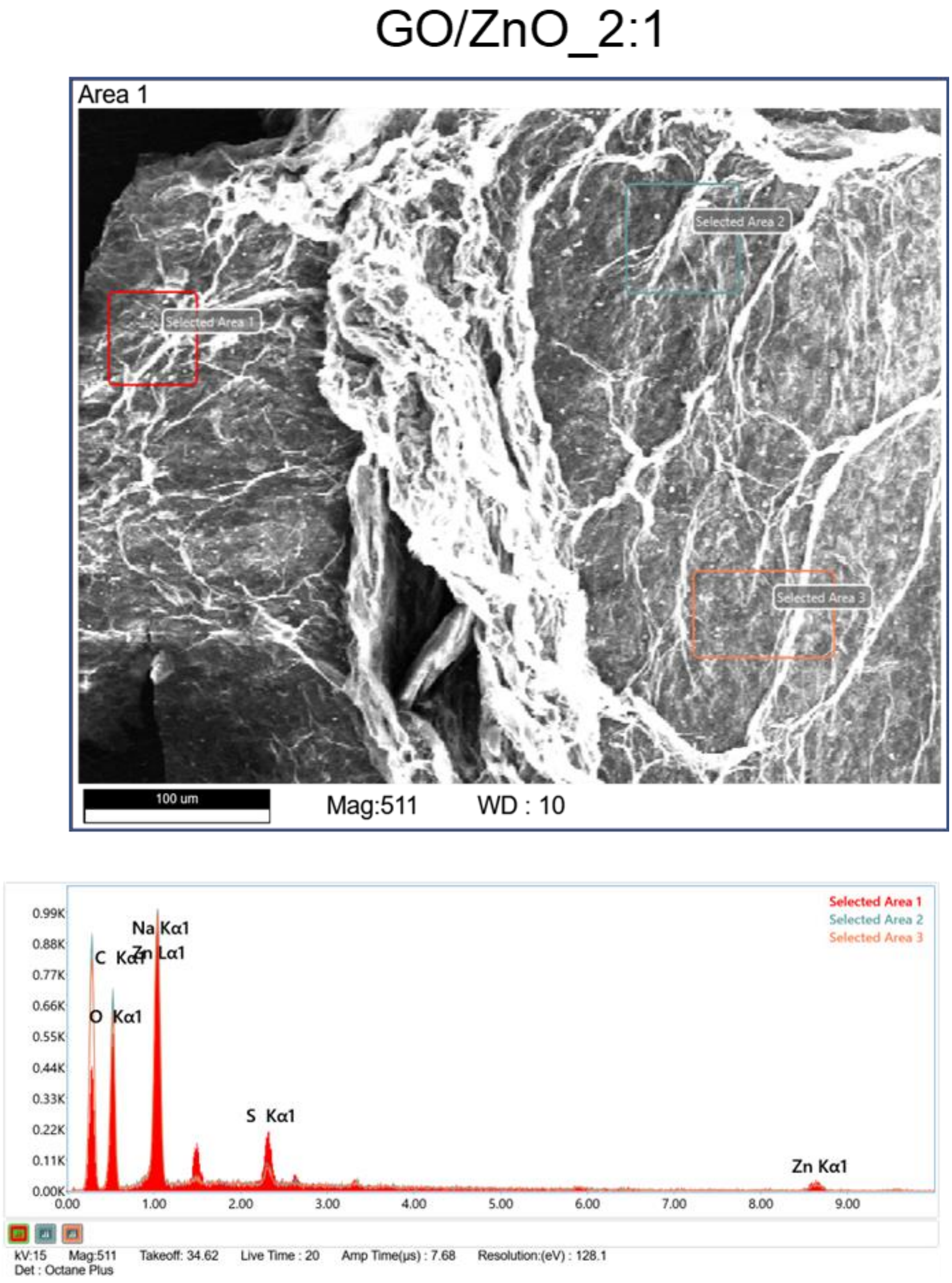

Figure S4. SEM pictures and element mapping results of GO/ZnO_2:1 composite.

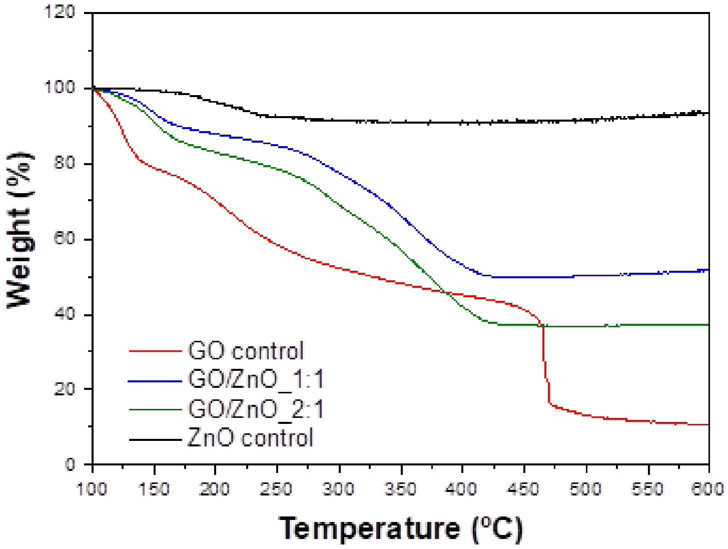

Figure S5. TGA analyses for GO, ZnO, GO/ZnO_1:1 and GO/ZnO_2:1 samples, performed under air atmosphere.

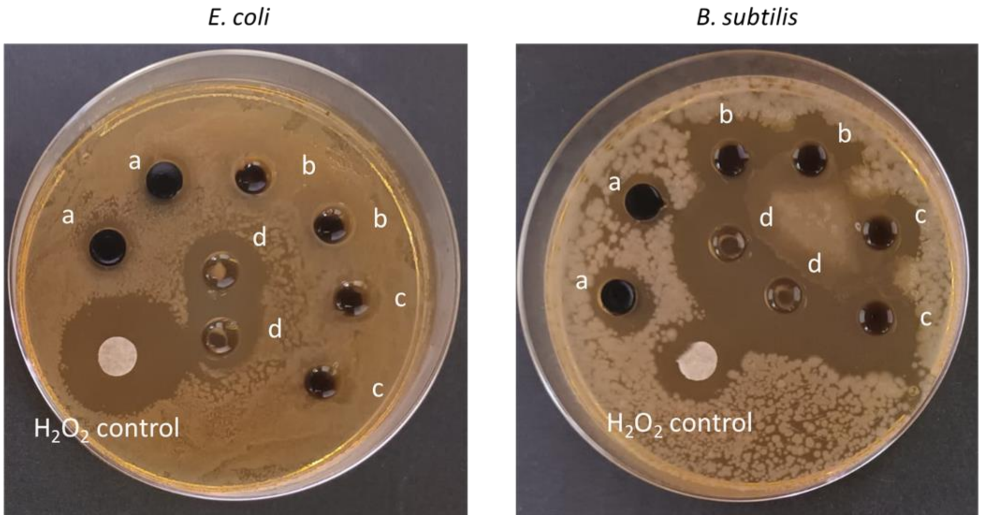

Figure S6. Antibacterial activity of GO (“a”, 10.0 mg/mL), GO/ZnO_1:1 (“b”, 0.1 mg/mL), GO/ZnO_2:1 (“c”, 0.1 mg/mL) and ZnO (“d”, 1.2 mg/mL) by the well diffusion assay against

Gram-negative (i.e., *E. coli,* left) and Gram-positive (i.e., *B. subtilis*, right) bacteria. Filter paper moistened with H_2O_2 was used as a positive control.

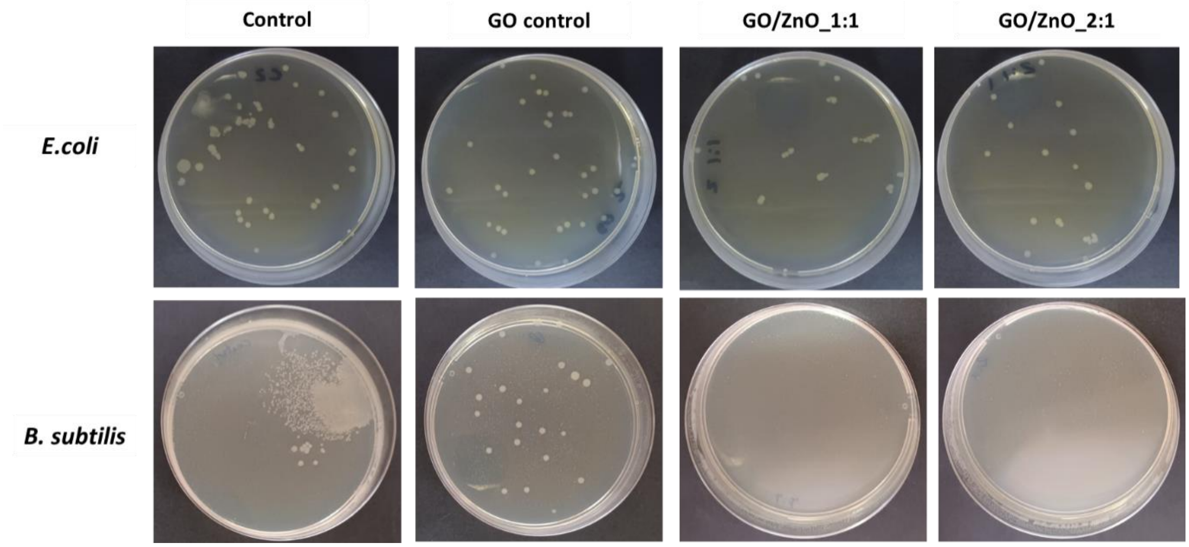

Figure S7. Representative digital images of the *E. coli* (upper panel) and *B. subtilis* (lower panel) culture plates after incubation in the presence of the different nanomaterials' dispersions (final concentration of 0.1 mg/mL). The control sample refers to incubation of both strains in the absence of any nanomaterial.

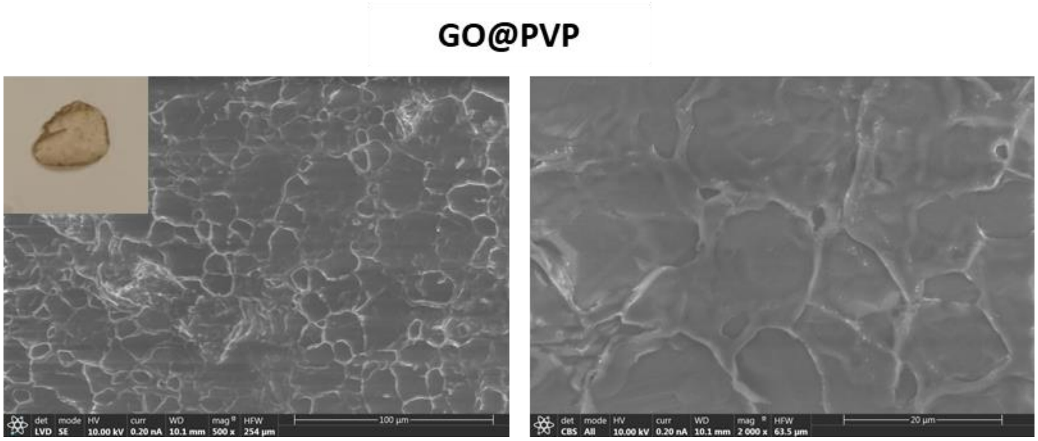

Figure S8. Representative SEM images of the GO@PVP control electrospun dressing at different magnifications. Scale bars: (left) 100 µm, (right) 20 µm. The inset image shows a digital photo of the dressing sample cut in round shapes with a diameter of 8 mm.

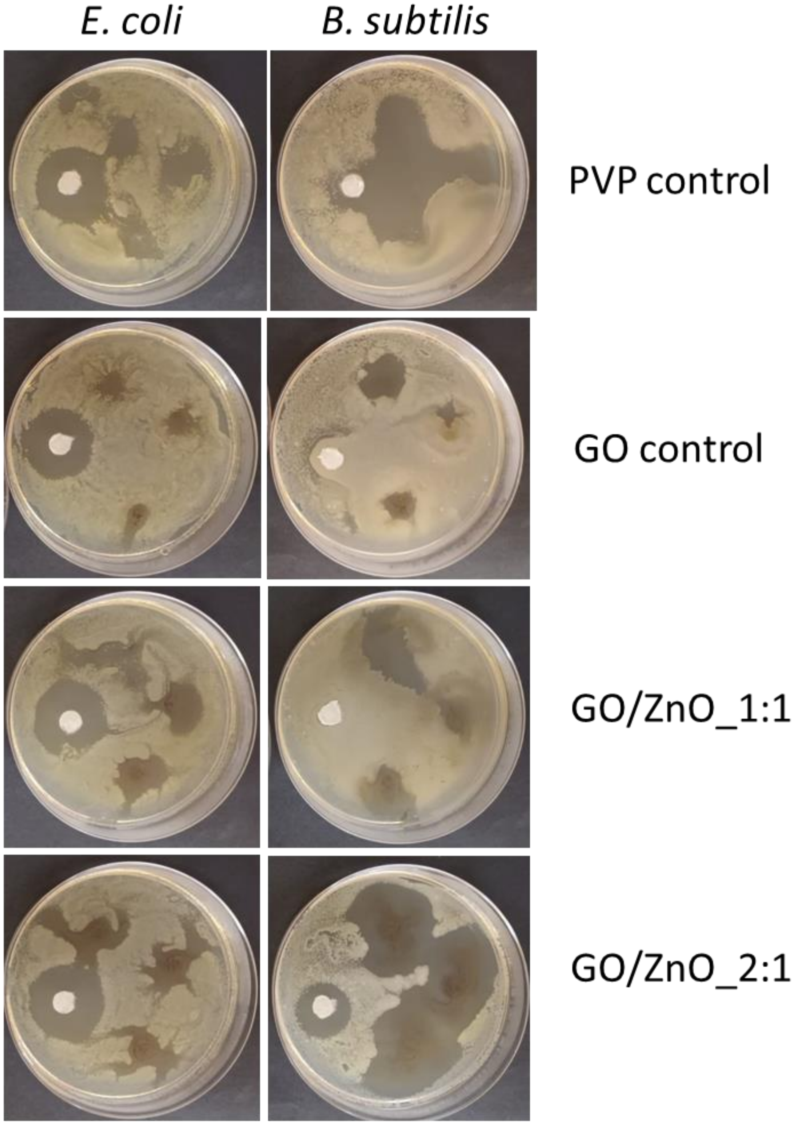

Figure S9. Digital images of *E. coli* (left panel) and *B. subtilis* (right panel) agar plates showing the three inhibition zones for each electrospun dressing sample after incubation at 37 ^{o}C. A positive control of H_2O_2 solution (1M) poured onto a round filter paper was added to each plate.
